# The Facial Nerve in Contemporary Surgery: Anatomical Variability, Pathology-Induced Distortion, and Functional Preservation

**DOI:** 10.3390/jcm15145622

**Published:** 2026-07-17

**Authors:** Piotr Łabętowicz, Nina Szczerba, Łukasz Olewnik, Nazar Włodarczyk, Kuba Borowski, Ingrid C. Landfald

**Affiliations:** 1 Department of Forensic Medicine, Pathology and Histology, Mazovian University in Płock, 09-402 Płock, Poland; p.labetowicz@mazowiecka.edu.pl; 2 VARIANTIS Research Laboratory, Mazovian University in Płock, 09-402 Płock, Poland; l.olewnik@mazowiecka.edu.pl; 3 Department of Clinical Anatomy, Mazovian University in Płock, 09-402 Płock, Poland; 4 VARIA Research Laboratory, Mazovian University in Płock, 09-402 Płock, Poland; 5 Department of Interventional Radiology, Medical University of Lodz, 90-419 Łódź, Poland; 6 Clinic of Orthopaedic and Paediatric Orthopaedics, Medical University of Lodz, 90-419 Łódź, Poland

**Keywords:** facial nerve, anatomical variation, vestibular schwannoma, hemifacial spasm, parotid surgery, facial reanimation

## Abstract

**Objectives:** The facial nerve (FN) possesses one of the most intricate anatomical courses in the head and neck, traversing the brainstem, temporal bone, and parotid gland before terminating within the muscles of facial expression. Owing to its complex anatomy, marked anatomical variability, and frequent distortion by adjacent pathology, preservation of FN integrity remains a fundamental challenge in skull base, otologic, and head and neck surgery. This review aims to provide a comprehensive synthesis of the contemporary literature regarding the clinical anatomy of the FN and to examine how anatomical variation, pathology-induced distortion, surgical strategy, and emerging technologies influence nerve preservation and functional outcomes. **Methods:** A comprehensive narrative review of the literature was conducted using PubMed, Scopus, and Google Scholar. Publications from 1983 through 2026 were searched using combinations of keywords, including “facial nerve,” “facial nerve anatomy,” “anatomical variation,” “vestibular schwannoma,” “hemifacial spasm,” “parotid surgery,” “facial nerve injury,” “facial nerve reconstruction,” “facial reanimation,” “diffusion tensor imaging,” “intraoperative neurophysiological monitoring,” and “artificial intelligence.” Peer-reviewed anatomical, radiological, clinical, and review articles published in English were included, while conference abstracts and studies lacking direct anatomical or surgical relevance were excluded. Particular emphasis was placed on surgically relevant anatomical variations, pathology-related anatomical distortion, advanced imaging modalities, intraoperative neurophysiological monitoring, reconstructive techniques, and predictors of postoperative facial nerve function. **Results:** Facial nerve preservation was found to depend on the interplay between individual anatomical variability, disease-related anatomical distortion, and operative strategy. In vestibular schwannoma surgery, nerve displacement, capsular adhesion, and cystic tumor degeneration were consistently associated with increased surgical complexity and less favorable postoperative facial function. In hemifacial spasm, successful microvascular decompression relied on precise identification of neurovascular conflict at the root exit zone. Within the parotid gland, substantial variability in branching architecture and surgical landmarks contributed to an increased risk of iatrogenic injury. Advanced imaging techniques, particularly diffusion tensor imaging tractography, improved preoperative prediction of FN location, while intraoperative neurophysiological monitoring enabled real-time assessment of neural integrity and functional preservation. Emerging artificial intelligence-based predictive models demonstrated potential to enhance patient-specific surgical planning and prognostication. **Conclusions:** Contemporary facial nerve surgery has evolved toward an individualized, anatomy-driven, and function-preserving paradigm supported by advanced imaging, intraoperative monitoring, and reconstructive strategies. Detailed understanding of both normal FN anatomy and pathology-induced anatomical distortion remains essential for optimizing surgical decision-making, maximizing nerve preservation, and improving long-term functional outcomes. Future developments integrating multimodal imaging, predictive analytics, and artificial intelligence may further refine patient-specific management and enhance postoperative facial function.

## 1. Introduction

### 1.1. Clinical and Functional Consequences of Facial Nerve Injury

The facial nerve (FN; cranial nerve VII) is one of the most anatomically complex and surgically vulnerable structures of the head and neck. Originating at the pontomedullary junction, it traverses the temporal bone through a series of narrow osseous canals before exiting the skull via the stylomastoid foramen and branching extensively within the parotid gland to innervate the muscles of facial expression. This long and intricate course places the nerve in close proximity to numerous neurovascular structures, making it particularly susceptible to a wide range of pathological processes [1].

Facial nerve injury, whether resulting from tumors, vascular compression, trauma, or surgical intervention, can lead to profound functional impairment and significant psychosocial morbidity. Facial paralysis compromises several essential functions, including eyelid closure, oral competence, speech articulation, and coordinated facial expression. Inadequate eyelid closure may result in corneal exposure and ocular injury, whereas dysfunction of the oral sphincter can impair speech and oral intake [2,3]. Beyond these functional deficits, the loss of facial symmetry and dynamic expression profoundly affects nonverbal communication and social interaction. Consequently, many patients experience social withdrawal, diminished self-confidence, and a substantial reduction in quality of life [4,5].

Preservation of facial nerve function is therefore a primary objective across multiple surgical specialties, including skull base, otologic, and head and neck surgery. Procedures performed in close proximity to the nerve require careful balancing of complete pathological treatment with maximal preservation of neural integrity. When preservation is not possible, timely nerve reconstruction and facial reanimation become essential for restoring both facial symmetry and dynamic facial function.

### 1.2. Anatomical Complexity and Surgical Vulnerability

Successful management of facial nerve pathology depends on a thorough understanding of its anatomical relationships. The nerve follows a complex course through both its intratemporal and extratemporal segments, giving rise to multiple branches whose configuration varies considerably among individuals. Variations in branching patterns, deviations in the course of the main trunk, and differences in the relationship between the nerve and key surgical landmarks have all been extensively documented [6,7,8].

In addition to congenital anatomical variations, the course of the facial nerve may be substantially altered by adjacent pathological processes. Vestibular schwannomas, parotid neoplasms, and other lesions may displace the nerve from its expected position, attenuate its fascicular architecture, or flatten its fibers along the tumor surface. These alterations frequently obscure conventional surgical landmarks and increase the risk of inadvertent intraoperative injury. Consequently, successful identification and preservation of the facial nerve require not only a detailed understanding of normal anatomy but also an appreciation of the ways in which pathological processes distort anatomical relationships.

Recognition of these anatomical challenges has fundamentally changed contemporary surgical practice. Whereas earlier approaches often prioritized complete lesion removal despite the risk of postoperative neurological deficits, modern strategies increasingly emphasize preservation of neural function through refined microsurgical techniques, meticulous dissection, and the integration of advanced imaging with intraoperative neurophysiological monitoring. Nerve-sparing tumor resection, staged treatment of selected benign lesions, and routine intraoperative monitoring have therefore become integral components of contemporary facial nerve surgery [9,10].

Accordingly, successful facial nerve surgery now depends on individualized anatomical interpretation that integrates microsurgical anatomy, pathology-induced distortion, preoperative imaging, and intraoperative functional assessment.

### 1.3. Aim of the Review

Despite the extensive body of literature addressing individual aspects of FN anatomy and surgery, including anatomical branching patterns, vestibular schwannoma surgery, microvascular decompression for hemifacial spasm, traumatic nerve injury, and parotid gland surgery, these topics have largely been studied independently. As a result, relatively few reviews provide a comprehensive synthesis that integrates anatomical variability, pathology-induced distortion, surgically relevant landmarks, operative strategies, predictors of postoperative functional outcomes, and emerging technological advances within a single clinically relevant framework. Since these interrelated factors collectively influence surgical planning, intraoperative decision-making, and the preservation of facial nerve function, a comprehensive review of the current evidence is warranted.

The aim of this review is to examine the clinical anatomy of the facial nerve and its implications for contemporary surgical management across a broad range of conditions, including vestibular schwannoma, hemifacial spasm, traumatic injury, and parotid tumors. Particular emphasis is placed on anatomical variation, surgically relevant landmarks, pathology-related distortion, predictors of postoperative facial nerve function, and the evolving role of advanced imaging, intraoperative neurophysiological monitoring, and emerging technologies in facilitating accurate nerve identification and preservation.

By integrating evidence from the anatomical, otologic, skull base, and head and neck surgery literature, this review provides a clinically relevant and anatomically grounded perspective on the factors that influence facial nerve preservation. It also offers a practical framework to support surgical planning, guide intraoperative decision-making, and optimize long-term functional outcomes.

## 2. Clinical Anatomy of the Facial Nerve

### 2.1. Central Origin and Intratemporal Course

The FN has both a deep (functional) and superficial (apparent) origins. Its deep origin comprises several brainstem nuclei that give rise to its motor, parasympathetic, special sensory, and general somatic sensory components. Motor fibers originate from the facial motor nucleus in the caudal pons, parasympathetic fibers arise from the superior salivatory nucleus, taste fibers project to the nucleus tractus solitarius, and general somatic sensory fibers terminate in the spinal trigeminal nucleus. The nerve emerges superficially at the pontomedullary junction as two roots: a larger motor root and a smaller mixed root, known as the nervus intermedius, which contains sensory, parasympathetic, and taste fibers [11,12].

Following its apparent origin, the FN traverses the cerebellopontine angle cistern within the subarachnoid space before entering the internal acoustic meatus (IAM). Throughout its intracranial cisternal course, the FN remains closely associated with the vestibulocochlear nerve (CN VIII). Within the IAM, it typically occupies the anterosuperior quadrant relative to the cochlear and vestibular nerves. The root exit zone represents the transition between central and peripheral myelin and is the principal site of neurovascular compression in hemifacial spasm, most commonly involving the anterior inferior cerebellar artery.

After entering the temporal bone through the IAM, the FN continues as the labyrinthine segment (LS), the shortest, narrowest, and one of the most vulnerable portions of its intratemporal course. This segment extends to the geniculate ganglion, located at the first genu of the facial canal, where the nerve makes an abrupt posterior turn into the tympanic segment. The greater petrosal nerve arises from the geniculate ganglion and carries preganglionic parasympathetic fibers to the lacrimal gland and the mucosal glands of the nasal cavity and palate. Owing to the confined anatomy of the facial canal, the labyrinthine segment and geniculate ganglion are particularly susceptible to inflammation-related compression associated with idiopathic facial paralysis (Bell’s palsy). They also serve as important anatomical landmarks during temporal bone and lateral skull base surgery.

Distally, the FN continues as the tympanic segment, coursing along the medial wall of the tympanic cavity adjacent to the oval window and lateral semicircular canal [13]. Before exiting through the stylomastoid foramen, the nerve descends within the mastoid (vertical) segment, which is located in the posterior wall of the tympanic cavity.

### 2.2. Extratemporal Course and Parotid Plexus

**Figure 1:**
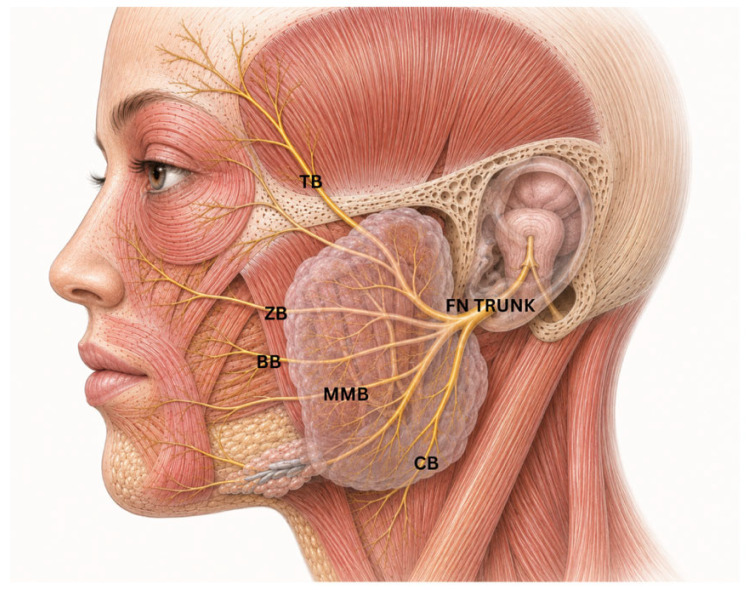
Extracranial course and intraparotid branching pattern of the facial nerve (FN) following its emergence from the stylomastoid foramen. The terminal branches include the temporal (TB), zygomatic (ZB), buccal (BB), marginal mandibular (MMB), and cervical (CB) branches. The parotid gland is shown as semi-transparent to illustrate the intraparotid neural plexus.

After exiting the temporal bone through the stylomastoid foramen, the FN gives rise to the nerve to the posterior belly of the digastric muscle and the nerve to the stylohyoid muscle [14,15]. It then enters the parotid gland, where it typically divides into two primary divisions, the temporofacial and cervicofacial trunks, which together form the parotid plexus (Figure 1) [16]. Although this bifurcation represents the classical anatomical configuration, considerable interindividual variation has been reported in the level of division, branching pattern, and presence of interbranch communications, making the intraparotid course of the FN highly variable [17].

Within the parotid gland, the FN further divides into five major terminal branches: the temporal, zygomatic, buccal, marginal mandibular, and cervical branches. These branches provide motor innervation to the muscles of facial expression. Owing to the complex organization of the parotid plexus, this region exhibits substantial anatomical variability, particularly with respect to branching patterns and interbranch communications. Such variability has important surgical implications, as unexpected branching configurations may complicate nerve identification during parotidectomy and increase the risk of inadvertent iatrogenic injury [18].

### 2.3. Surgically Relevant Landmarks

Parotid tumors are the most common neoplasms of the salivary glands, and parotidectomy remains the treatment of choice for most lesions. Accurate identification of surgically relevant FN landmarks is therefore essential to minimize the risk of iatrogenic nerve injury during surgery. However, anatomical variability and tumor-related distortion may reduce the reliability of individual landmarks, often necessitating the use of multiple complementary anatomical references during surgical dissection.

Bony landmarks are generally considered the most reliable because of their relatively constant anatomical position. Among these, identification of the FN in relation to the TMP is regarded as particularly reliable owing to the ease with which this landmark can be palpated [18]. Another important landmark is the tragal pointer (TP), which corresponds to the region where the FN emerges superior to the posterior belly of the digastric muscle and adjacent to the tympanomastoid suture (TMS) line. The retromandibular vein represents a softer and less consistent anatomical landmark; nevertheless, it remains clinically useful during parotid surgery. In most individuals, the temporofacial and cervicofacial divisions of the FN course superficial and lateral to the retromandibular vein [19]. Consequently, contemporary parotid surgery relies on a combination of fixed osseous landmarks, soft tissue landmarks, and meticulous microsurgical dissection rather than on any single anatomical landmark.

## 3. Anatomical Variations in the Facial Nerve

### 3.1. Variations in Branching Pattern

The extratemporal branching pattern of the FN within the parotid gland exhibits considerable anatomical variability and has therefore been the subject of extensive anatomical and surgical investigation. This variability is of particular clinical importance because accurate identification and preservation of the facial nerve are fundamental objectives during parotidectomy. The presence of parotid tumors may further distort the normal branching architecture, reducing the reliability of expected anatomical relationships and increasing the complexity of surgical dissection. Consequently, atypical trunk configurations, complex plexiform branching patterns, and numerous interbranch communications may complicate intraoperative nerve identification and increase the risk of inadvertent facial nerve injury [18].

**Figure 2:**
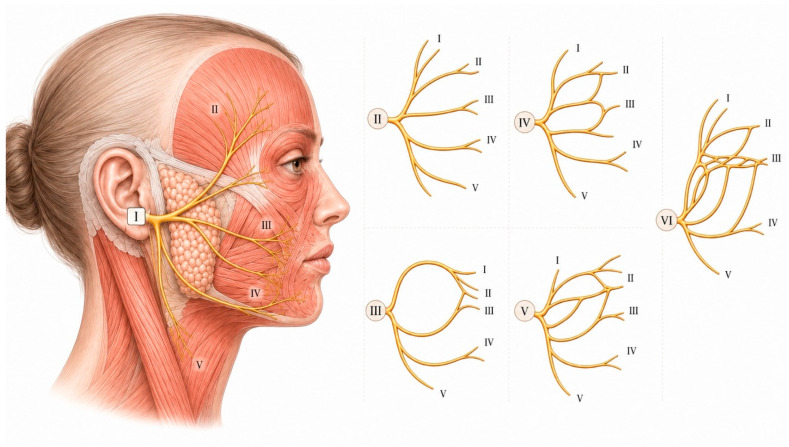
Davis classification of the extratemporal branching patterns of the facial nerve within the parotid gland. Six major types (I–VI) are illustrated based on the degree of anastomotic communications between the temporal, zygomatic, buccal, marginal mandibular, and cervical branches forming the parotid plexus (pes anserinus)—schematic illustration created by the authors based on the classification described by Davis et al. [20].

The classification proposed by Davis et al. in 1956 [20] remains the most widely recognized system for describing the intraparotid branching architecture of the facial nerve. Based on the presence, distribution, and complexity of anastomotic communications between the terminal branches, the parotid plexus is categorized into six branching patterns (Types I–VI) (Figure 2) [18].

A recent systematic review and meta-analysis demonstrated that Type III represents the most prevalent branching pattern (23.1%), followed by Types IV (19.9%), II (17.3%), and I (15.5%) [21]. Furthermore, bifurcation of the facial nerve trunk into temporofacial and cervicofacial divisions is observed in approximately 94.1% of individuals, whereas trifurcation represents an uncommon anatomical variant. These findings emphasize that substantial anatomical variability should be anticipated during parotidectomy rather than assuming a single standard branching configuration [21,22].

**Figure 3:**
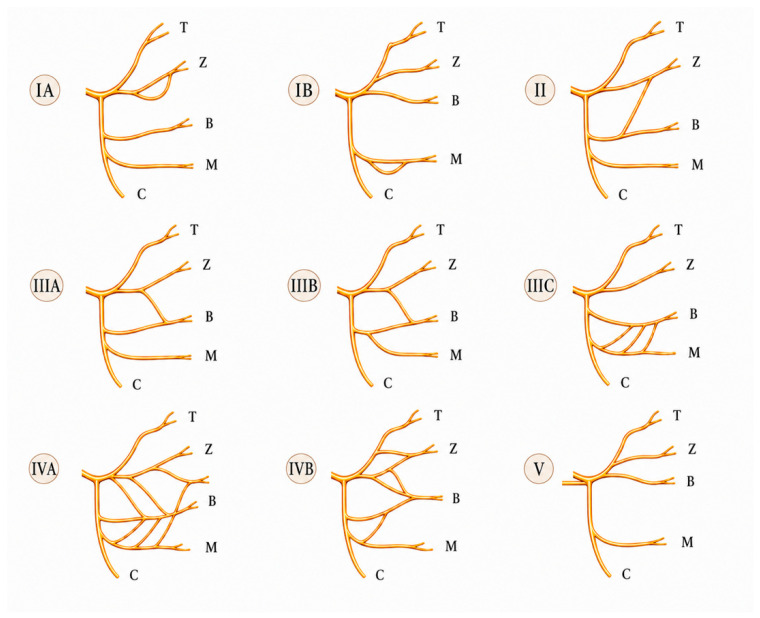
Katz and Catalano classification of the extratemporal facial nerve branching patterns within the parotid gland, including the additional double-trunk variants described by Kopuz et al. (VA–VC). Schematic illustration created by the authors based on the classifications proposed by Katz and Catalano and modified by Kopuz et al. [23].

To provide a more detailed description of extratemporal facial nerve anatomy, Katz and Catalano introduced an expanded classification in 1987 consisting of nine branching patterns (IA, IB, II, IIIA, IIIB, IIIC, IVA, IVB, and V), distinguished by the origin of the buccal branch, the pattern of interbranch communications, and the number of main nerve trunks (Figure 3). Kopuz et al. subsequently modified this classification by introducing three additional double-trunk variants (VA–VC), further highlighting the complexity of extratemporal facial nerve anatomy [23].

In the original Katz and Catalano series, Type III was the most frequently encountered pattern (44%), followed by Type I (24%), Types II and IV (14% each), whereas Type V was identified in only 3% of specimens. Although this classification has been adopted less frequently than the Davis system, it provides a more comprehensive description of branching morphology and may be particularly useful when anticipating complex intraparotid anatomy during parotidectomy [21,22].

Beyond these recognized classification systems, numerous studies have demonstrated that the “classical” branching pattern is absent in a substantial proportion of individuals. Instead, a complex plexiform arrangement with extensive interbranch communications is frequently encountered. These communications may occur between the temporofacial and cervicofacial divisions or between individual terminal branches, most commonly involving the buccal branch and either the marginal mandibular or zygomatic branches [22,24,25]. Collectively, these findings demonstrate that no single branching pattern can be considered universally representative. Consequently, familiarity with both common and uncommon branching configurations is essential for safe parotidectomy and other surgical procedures involving the parotid region [24,25,26].

### 3.2. Variability of Surgical Landmarks

In addition to variability in branching patterns, the anatomical relationships between the facial nerve trunk and commonly used surgical landmarks also demonstrate considerable interindividual variation. Accurate identification of these landmarks is essential during parotidectomy, particularly when the facial nerve cannot be visualized directly.

Among the most frequently used landmarks, the posterior belly of the digastric muscle and the TMS have been shown to be relatively consistent, whereas the tragal pointer demonstrates greater anatomical variability [27,28]. Reported differences are influenced by factors such as age, sex, and individual anatomical variations.

In a cadaveric study by Stankevicius and Suchomlinov [22], the mean distance between the facial nerve trunk and the tragal pointer was 9.30 ± 0.93 mm (range, 7.67–10.78 mm). The mean distance between the facial nerve trunk and the angle of the mandible measured 36.45 ± 4.14 mm (range, 25.84–44.39 mm), whereas the distance to the TMS was 12.53 ± 2.30 mm (range, 8.99–17.26 mm). These findings reinforce the importance of using multiple anatomical landmarks rather than relying on a single reference point during facial nerve identification.

### 3.3. Communication with Adjacent Nerves

The FN also demonstrates important communication with adjacent cranial nerves within and around the parotid gland. The auriculotemporal nerve, a branch of the mandibular division of the trigeminal nerve (CN V), frequently communicates with branches of the facial nerve in the parotid region [29].

Additional communications have been described between the facial nerve and the glossopharyngeal nerve (CN IX) within the parotid gland and adjacent anatomical regions. Although these neural connections are relatively uncommon, they are clinically relevant during parotid and skull base surgery because they may increase the risk of inadvertent nerve injury and contribute to atypical postoperative neurological findings. Recognition of these anatomical communications may therefore facilitate safer surgical dissection and improve the interpretation of unexpected postoperative sensory or motor deficits.

The clinically relevant anatomical variations of the extratemporal facial nerve are summarized in Table 1.

## 4. Anatomical Distortion in Disease

### 4.1. Vestibular Schwannomas

Vestibular schwannomas (VS), also known as acoustic neurinomas, are the most common tumors of the cerebellopontine angle. As the tumor grows along the capsule, the adherences between it and the FN start to form, which leads to stretching and flattening of the nerve [30]. VSs’ most frequent location is in the posterior vestibular nerves, causing FNs anterior, anterosuperior or anteroinferior display [31]. The tumor may also be located within the internal auditory canal or the cerebellopontine angle, placing the FN on the inferior aspect of the tumor [10]. Beyond tumor size, the tumor adhesions and the nerve placement are critical predictors of postoperative facial function [32].

### 4.2. Hemifacial Spasm

The rare movement disorder known as hemifacial spasm (HS) is characterized by involuntary unilateral contractions of the muscles of facial expression innervated by the FN. The condition occurs more frequently on the left side and demonstrates a higher prevalence in women than in men.

Current evidence indicates that neurovascular compression at the facial nerve root exit zone is the primary etiological mechanism underlying HS. The most common offending vessels are the anterior inferior cerebellar artery (AICA) and posterior inferior cerebellar artery (PICA), accounting for approximately 40.8% and 24.9% of cases, respectively. Combined compression by both arteries has been reported in approximately 26.8% of patients. Although HS is not a life-threatening condition, symptoms such as involuntary eye closure may significantly impair quality of life and contribute to functional visual disturbances [33,34,35].

### 4.3. Traumatic and Iatrogenic Injury

Depending on the type of trauma or surgical intervention, different segments of the FN may be vulnerable to injury. Temporal bone fractures and other craniofacial trauma most commonly affect the intratemporal segment, resulting in facial nerve paresis or paralysis. The incidence of FN dysfunction is significantly higher in transverse temporal bone fractures compared with longitudinal fractures [36].

The extratemporal segment of the FN is particularly susceptible to injury during parotid surgery. Following parotidectomy or other parotid trauma, aberrant regeneration of parasympathetic fibers originating from the glossopharyngeal nerve (CN IX) via the auriculotemporal nerve may result in erythema and gustatory sweating, a condition known as Frey syndrome or auriculotemporal syndrome [37].

Key disease-related anatomical distortions of the facial nerve are summarized in Table 2.

## 5. Surgical Implications of Facial Nerve Anatomy

The anatomical course of the FN directly influences surgical strategy in procedures involving the cerebellopontine angle, temporal bone, and parotid region. Adjacent pathology frequently distorts the normal anatomical position of the nerve, thereby increasing the risk of iatrogenic injury and postoperative functional impairment. Successful management therefore depends on accurate anatomical orientation, meticulous microsurgical dissection, and appropriate reconstructive strategies when preservation of the FN is not feasible.

### 5.1. Vestibular Schwannoma Surgery

VS frequently displace, stretch, or thin the FN along the tumor capsule, making preservation technically challenging [38]. Despite these difficulties, contemporary microsurgical techniques achieve favorable functional outcomes corresponding to House–Brackmann grade I–II in approximately 70–96% of patients. However, this wide range reflects considerable heterogeneity among the available studies, including differences in tumor size, surgical approach, patient selection, follow-up duration, and outcome assessment [37,38,39,40].

Successful preservation depends on meticulous capsular dissection, minimization of traction injury, and the use of intraoperative neurophysiological monitoring, particularly in large or anatomically distorted tumors [41]. In selected cases, subtotal resection followed by stereotactic radiosurgery may further improve functional preservation while maintaining satisfactory long-term tumor control [42].

### 5.2. Microvascular Decompression for Hemifacial Spasm

Hemifacial spasm results from vascular compression of the FN at the root exit zone. Microvascular decompression remains the standard surgical treatment and provides long-term symptom relief in more than 90% of patients [43,44].

Successful decompression depends on accurate identification of the offending vessel and adequate separation of the neurovascular conflict. The shelter technique, which repositions the compressing vessel away from the nerve, has demonstrated higher cure rates compared with traditional interposition techniques [45]. Intraoperative monitoring using blink reflex assessment and lateral spread response analysis may further help confirm adequate decompression and optimize postoperative functional outcomes [42,46,47].

### 5.3. Parotidectomy Techniques

Within the parotid gland, the FN forms a highly variable branching network that is particularly vulnerable during tumor resection. Identification of the main trunk and meticulous dissection along peripheral branches are therefore essential to minimize the risk of postoperative facial palsy.

Retrograde dissection, in which peripheral branches are followed proximally toward the main trunk, has been associated with lower rates of temporary facial weakness compared with the traditional anterograde approach [48]. In selected benign lesions, extracapsular dissection may further reduce unnecessary nerve manipulation while maintaining oncological safety [49].

### 5.4. Facial Nerve Repair and Grafting

When preservation of the FN is not possible, reconstructive procedures are required to restore neural continuity and improve postoperative facial function. Direct end-to-end neurorrhaphy remains the preferred technique when tension-free approximation of the nerve stumps can be achieved. When direct repair is not feasible, interposition nerve grafting is most commonly performed.

Functional recovery corresponding to House–Brackmann grade IV or better has been reported in approximately 68–79% of patients undergoing nerve graft reconstruction [47,50]. Outcomes are generally more favorable following traumatic injury than after tumor-related resection. Furthermore, early surgical repair, ideally within six months of injury, significantly improves the likelihood of successful reinnervation and long-term functional recovery [51].

### 5.5. Nerve Transfer and Reanimation

In cases of long-standing facial paralysis or absence of a proximal FN stump, nerve transfer procedures represent an important strategy for facial reanimation. The most commonly performed techniques include hypoglossal–facial and masseteric–facial nerve transfers. These procedures achieve functional recovery corresponding to House–Brackmann grade III in approximately 60–70% of patients [52]. Modified hypoglossal–facial techniques preserving partial hypoglossal continuity have been shown to significantly reduce tongue morbidity while maintaining effective facial reinnervation. In more complex cases, nerve transfer procedures may additionally be combined with cross-face nerve grafting or free functional muscle transfer to restore dynamic facial expression and improve facial symmetry [53]. Among free functional muscle transfers, the gracilis muscle remains the most widely used donor muscle because of its reliable neurovascular anatomy, favorable excursion and predictable functional outcomes in facial reanimation. Detailed understanding of the surgical anatomy of the masseter muscle and its anatomical variations is essential for safe dissection and successful nerve transfer [54,55,56].

The principal anatomical determinants of facial nerve preservation across contemporary surgical procedures are summarized in Table 3.

## 6. Predictors of Functional Outcome and Technological Advances in FN Surgery

### 6.1. Predictors of Outcome and Preoperative Mapping of the FN

Postoperative functional outcome of the FN depends on multiple interacting factors. The most important predictors include the anatomical relationship between the nerve and the underlying pathology, intraoperative findings, as well as patient- and tumor-related characteristics.

The anatomical position of the FN is particularly important in conditions such as VS, where tumor growth along the capsule may displace, stretch, or thin the nerve. Large tumors with strong adhesions to the FN and/or cystic consistency often require greater surgical manipulation and are therefore associated with less favorable postoperative outcomes, frequently resulting in House–Brackmann grade III or worse facial function [32,61].

In addition, fibrous tumor stroma may increase operative difficulty by limiting effective vascular coagulation and reducing dissection planes between the tumor capsule and adjacent neural structures [61].

Given the significant anatomical variability of the FN, preoperative mapping plays an increasingly important role in surgical planning. MRI may be used both preoperatively and intraoperatively to assess not only tumor consistency, but also FN displacement, distortion, or thinning caused by adjacent pathology [62]. MRI may additionally demonstrate the presence of cerebrospinal fluid within the lateral internal auditory canal, including the so-called fundal fluid cap, which has been associated with improved clinical outcomes, particularly preservation of hearing in small intracanalicular VS [63].

Another important tool in preoperative planning is diffusion tensor imaging tractography (DTIT). Because water diffusion within white matter pathways exhibits anisotropic properties, DTIT allows three-dimensional reconstruction of neural pathways and may help estimate the course of the FN around tumor margins with relatively high accuracy [64]. This is particularly valuable in large VS, where substantial anatomical distortion may reduce the reliability of conventional anatomical expectations.

Patient-related factors also appear to influence postoperative FN recovery. Younger age at surgery has been associated with more favorable postoperative functional outcomes and higher likelihood of complete recovery [65]. Collectively, these findings suggest that integration of anatomical assessment, advanced imaging techniques, tumor characteristics, and patient-specific factors may substantially improve surgical planning and contribute to more favorable postoperative FN preservation.

### 6.2. Intraoperative Neurophysiological Monitoring

Intraoperative neurophysiological monitoring is as important as preoperative mapping because it provides real-time feedback on facial nerve location and functional integrity, thereby reducing the risk of iatrogenic injury. During microvascular decompression for hemifacial spasm, the LSR is used intraoperatively to predict postoperative symptom remission. Disappearance of the LSR following successful decompression is generally considered indicative of adequate relief of neurovascular compression [66]. Another important monitoring modality is blink reflex monitoring, which evaluates the functional integrity of both the facial nerve (CN VII) and the trigeminal nerve (CN V). Intraoperative corneal stimulation activates mechanoreceptors and free nerve endings within the corneal epithelium, transmitting afferent impulses through the nasociliary nerve and the ophthalmic division of the trigeminal nerve to the principal sensory nucleus of the trigeminal nerve in the rostral pons. Interneurons then project bilaterally to the facial motor nuclei, with efferent fibers traveling through the temporal and zygomatic branches of the facial nerve to the orbicularis oculi muscles, resulting in synchronous eyelid closure. Blink reflex monitoring serves as an important predictor of postoperative facial nerve function, particularly during posterior skull base surgery, and is commonly used in conjunction with EMG. The intensity of corneal stimulation may be gradually increased until a movement artifact is detected in the EMG recordings of either the ipsilateral or contralateral orbicularis oculi muscle [67].

### 6.3. Artificial Intelligence and Predictive Models

AI is emerging as a promising adjunct in facial nerve surgery, with potential applications in preoperative planning, intraoperative decision-making, and the prediction of postoperative functional outcomes. Rather than replacing surgical judgment or anatomical expertise, AI may assist surgeons by integrating multidimensional datasets to support individualized surgical planning, particularly during complex procedures.

Unlike conventional prognostic approaches, which typically rely on isolated clinical or anatomical factors, AI can integrate multidimensional datasets to identify complex relationships between patient-, pathology-, imaging-, and surgery-related variables. Machine learning algorithms can detect nonlinear interactions that may improve prediction of postoperative FN outcomes and support individualized risk assessment. AI has also shown potential in preoperative surgical planning, clinical decision support, and the interpretation of intraoperative monitoring data, including free-running electromyography (EMG) and LSR recordings. However, current applications remain investigational, and further prospective validation is required before AI can be routinely implemented in clinical practice [64,65].

A comprehensive summary of these contemporary determinants, along with their clinical influences and current limitations, is detailed in Table 4.

## 7. Complications and Long-Term Outcomes

### 7.1. Postoperative Complications

Despite substantial advances in FN surgery, postoperative FN dysfunction remains a significant source of morbidity following skull base, parotid, temporal bone, and other head and neck procedures performed in close proximity to the nerve. Complications may occur immediately after surgery or develop in a delayed fashion, depending on the underlying pathology, the extent of dissection, and the degree of neural manipulation.

Microvascular decompression for HS may result in acute postoperative FN palsy developing within the first 24 h after surgery. Although this condition is often transient, the average reported recovery time is approximately 85 days; however, the available evidence remains limited and heterogeneous [34].

Delayed facial weakness may also develop days to weeks after initially preserved postoperative FN function and is observed more frequently following VS surgery or parotidectomy [34]. Proposed mechanisms include postoperative edema, vascular compromise, traction injury, and delayed neural inflammation.

Another important long-term consequence of aberrant neural regeneration is synkinesis, in which voluntary movement of one facial region triggers involuntary contraction of another. Common manifestations include involuntary eye closure during smiling and unintended movement of the oral commissure during blinking [51,58].

Ocular complications represent some of the most functionally significant consequences of FN dysfunction. Major complications include lagophthalmos, defined as incomplete eyelid closure, and exposure keratopathy resulting from corneal desiccation. If left untreated, these conditions may lead to corneal ulceration, chronic ocular irritation, and visual impairment [69].

In addition, injury to the auriculotemporal nerve during parotidectomy may result in aberrant parasympathetic reinnervation, leading to gustatory sweating, flushing, and erythema within the preauricular and temporal regions, a condition known as Frey syndrome [37].

### 7.2. Long-Term Functional Recovery

Time to intervention remains one of the strongest predictors of long-term postoperative recovery. Early decompression or repair of the FN is associated with significantly better functional outcomes than delayed intervention. Early management of FN paralysis has been associated with postoperative recovery corresponding to House–Brackmann grade II, whereas delayed decompression is more frequently associated with poorer recovery, including House–Brackmann grade IV function [70].

Delayed repair is also associated with progressive muscular atrophy, fibrosis, and reduced reinnervation potential. In contrast, early reconstruction promotes neural regeneration and increases the likelihood of favorable long-term functional recovery [70].

## 8. Discussion

### 8.1. Anatomical Variability and Surgical Complexity

The evidence reviewed throughout this study demonstrates that modern FN surgery increasingly relies on interpretation of patient-specific and pathology-dependent anatomy rather than adherence to fixed anatomical expectations. Although classical descriptions of the FN and parotid plexus remain fundamentally important, substantial variability in branching patterns, interbranch communications, and relationships to adjacent structures may markedly influence operative complexity and postoperative functional outcomes [21,22,24,25,26]. These anatomical variations should therefore be viewed not simply as descriptive findings, but as clinically significant configurations capable of altering dissection planes, obscuring nerve identification, and increasing susceptibility to iatrogenic injury.

Within the parotid region, complex plexiform arrangements and communications between terminal branches may further complicate identification of individual branches during tumor resection and reconstructive procedures [22,24,25,26]. Likewise, variability involving the marginal mandibular, buccal, and zygomatic branches may substantially affect surgical exposure and postoperative facial symmetry [29]. Collectively, these observations demonstrate that “classic” branching models represent generalized anatomical patterns rather than universally reproducible intraoperative anatomy.

A similar principle applies to surgical landmarks. Although structures such as the TMS, posterior belly of the digastric muscle, mastoid process, tragal pointer, and retromandibular vein remain important intraoperative references, their reliability may decrease considerably in the setting of recurrent disease, inflammatory distortion, previous surgery, or large neoplastic lesions [27,28]. Consequently, current evidence strongly supports a multi-landmark strategy combined with continuous anatomical reassessment during dissection. Together, these findings reflect an important conceptual transition in FN surgery, where anatomy is increasingly interpreted dynamically rather than as a static and universally predictable configuration.

### 8.2. Pathology-Related Distortion of FN Anatomy

Another major theme identified throughout the reviewed literature is the substantial influence of pathology-related anatomical distortion on operative complexity and postoperative FN preservation. In VS surgery, functional outcomes appear to depend less on tumor size alone and more on the anatomical relationship between the lesion and the FN, including displacement pattern, degree of capsular adhesion, cystic degeneration, and thinning of neural structures [28,29,30]. These observations are particularly important because they indicate that conventional radiological measurements alone may inadequately predict surgical risk. Instead, the interaction between pathology and regional anatomy becomes a primary determinant of operative difficulty.

A similar principle is observed in HS, where the critical factor is not merely the presence of vascular compression, but the precise neurovascular configuration at the root exit zone [33,34,35]. Compression by the AICA, PICA, vertebral artery, or combined vascular loops may produce highly variable microsurgical anatomy that directly influences decompression strategy and postoperative remission rates [33,34,35]. Together, these findings emphasize that successful FN surgery depends on pathology-specific anatomical interpretation rather than reliance on generalized anatomical models.

The reviewed evidence additionally suggests that recurrent lesions represent a particularly demanding surgical subgroup because fibrosis, scarring, and loss of normal tissue planes substantially reduce the reliability of conventional orientation techniques. This is especially relevant in recurrent parotid pathology, where postoperative fibrosis may obscure the FN trunk and peripheral branches while increasing susceptibility to traction injury, incomplete nerve recognition, or inadvertent branch manipulation. Collectively, these findings support the concept that pathology-induced anatomical distortion is one of the principal contributors to FN morbidity across skull base and head and neck surgery.

### 8.3. Evolution Toward Function-Preserving Surgery

One of the most important developments identified throughout the literature is the ongoing transition from purely lesion-oriented surgery toward function-preserving surgical strategies. Historically, maximal resection frequently represented the primary objective even when associated with substantial neurological morbidity. Current management increasingly prioritizes long-term FN preservation and postoperative quality of life while maintaining acceptable oncological or symptomatic control [42].

This evolution is particularly evident in VS surgery, where subtotal resection followed by stereotactic radiosurgery has emerged as a valuable strategy in selected patients to reduce traction injury and preserve postoperative FN function [9,10]. Similarly, extracapsular dissection and retrograde dissection techniques in parotid surgery aim to minimize unnecessary nerve manipulation while maintaining oncological safety in appropriately selected lesions [48,49]. The increasing use of nerve-sparing techniques across multiple surgical disciplines reflects broader recognition that preservation of dynamic facial function represents a central determinant of surgical success rather than a secondary consideration.

Importantly, the reviewed evidence also emphasizes that successful FN management extends beyond anatomical continuity alone. Delayed palsy, synkinesis, ocular morbidity, oral incompetence, and long-term asymmetry may occur despite technically successful surgery [41,52,60,69]. Consequently, postoperative evaluation increasingly requires consideration of dynamic facial function, ocular protection, rehabilitation potential, and patient-reported quality of life rather than reliance solely on immediate postoperative grading systems.

When preservation is not feasible, reconstruction and reanimation become essential components of management. The literature demonstrates that outcomes following neurorrhaphy, interpositional grafting, hypoglossal-facial transfer, masseteric-facial transfer, and free functional muscle transfer remain highly dependent on timing and denervation duration [47,48,51,58]. Early intervention appears particularly important because prolonged denervation is associated with progressive muscular atrophy, fibrosis, and reduced reinnervation potential. These findings reinforce that FN surgery should be interpreted as a longitudinal reconstructive process extending beyond the intraoperative period.

### 8.4. Imaging, Intraoperative Monitoring, and Technological Integration

Another major finding of this review is the expanding integration of advanced imaging and intraoperative monitoring into FN surgery. MRI-based evaluation of tumor consistency, displacement patterns, and internal auditory canal morphology increasingly contributes to preoperative risk assessment and surgical planning [53,61,62]. Particularly important is the emergence of DTIT, which allows preoperative estimation of FN position around VS and represents a transition from generalized anatomical expectation toward patient-specific anatomical prediction [64].

A similar transition is evident in intraoperative neurophysiological monitoring. The increasing use of EMG, blink reflex monitoring, and LSR analysis reflects movement from static anatomical orientation toward real-time functional assessment during surgery [50,57,59,64,65].

In HS surgery, disappearance of the LSR has been strongly associated with adequate decompression and favorable postoperative remission [57,59]. However, the literature also suggests that monitoring should be interpreted as an adjunctive tool rather than a substitute for anatomical expertise. Neurophysiological data remain dependent on surgical context, interpretation, and technical limitations, emphasizing that optimal outcomes are most likely achieved when monitoring is integrated with detailed microsurgical anatomical understanding.

### 8.5. Artificial Intelligence and Future Surgical Planning

AI and predictive computational models represent an emerging direction in contemporary FN surgery. Machine learning systems may integrate anatomical, radiological, neurophysiological, and clinical variables to improve prediction of postoperative functional outcomes and support individualized surgical planning [67,68]. Unlike conventional prognostic assessment based on isolated predictors, AI-based models may identify complex nonlinear relationships that are difficult to detect manually.

However, current evidence remains limited by retrospective study designs, relatively small datasets, lack of external validation, and heterogeneity in imaging protocols, predictive algorithms, and outcome measures [67,68]. Consequently, AI should currently be regarded as a promising adjunctive tool that may enhance, but not replace, surgical judgment, anatomical expertise, and microsurgical experience.

### 8.6. Limitations and Future Directions

Despite substantial advances in FN surgery, the current literature remains limited by retrospective study designs, heterogeneity in pathology and surgical technique, variable follow-up duration, and inconsistent functional assessment. Reliance on House–Brackmann grading alone may additionally underestimate important postoperative outcomes, including synkinesis, ocular morbidity, facial symmetry, and patient-reported quality of life.

Further limitations involve the lack of standardized protocols for imaging, DTIT acquisition, and intraoperative neurophysiological monitoring. Although technological integration has improved considerably, many systems remain institution-dependent and lack universal reproducibility. Similarly, evidence regarding AI-assisted prediction remains preliminary and requires prospective multicenter validation before broader clinical implementation.

Future research should increasingly focus on patient-specific anatomy and multimodal surgical planning integrating advanced imaging, tractography, intraoperative monitoring, and predictive computational systems. Greater emphasis should also be placed on correlating anatomical variability with long-term functional outcomes, particularly in recurrent parotid pathology and complex skull base lesions.

Overall, the reviewed literature supports the concept that FN surgery is evolving from traditional landmark-based approaches toward individualized, anatomy-driven, technology-assisted functional preservation. The most favorable outcomes are therefore likely achieved when refined microsurgical technique is combined with detailed anatomical understanding, advanced imaging, intraoperative functional assessment, and patient-specific operative planning.

## 9. Conclusions

Contemporary FN surgery increasingly depends on individualized anatomical interpretation integrating microsurgical anatomy, pathology-related distortion, advanced imaging, and intraoperative neurophysiological monitoring. Although classical anatomical landmarks remain fundamental, substantial anatomical variability and disease-induced distortion may significantly alter surgical orientation, operative complexity, and postoperative functional outcomes.

Current evidence supports a continued transition from purely lesion-oriented surgery toward function-preserving and reconstructive strategies emphasizing long-term facial function and quality of life in addition to disease control. Emerging technologies, including DTIT and AI-assisted predictive models, further contribute to patient-specific surgical planning and dynamic intraoperative assessment.

Ultimately, successful FN management requires integration of refined microsurgical technique with detailed anatomical understanding, advanced technological support, and longitudinal functional rehabilitation. As surgical practice continues to evolve, preservation of dynamic facial function will likely remain one of the principal determinants of successful treatment across skull base and head and neck surgery.

## Figures and Tables

**Table 1:** Clinically relevant variability of the extratemporal facial nerve.

Variable Feature	Reported Prevalence	High-Yield Finding
Branching pattern	Classical configuration ~15–16% [21]	“Classic” FN configuration is frequently absent
Davis classification (I–VI)	Type III 23.1%; Type IV 19.9%; Type II 17.3%; Type I 15.5% [21]	Increasing interbranch anastomoses with higher types
Katz–Catalano classification	Type III 44%; Type I 24%; Types II and IV 14% each; Type V 3% [21]	Greater variability in buccal branch origin and trunk organization
Double-trunk variants	Uncommon anatomical variant [21,22]	Duplication of the main FN trunk may occur
Plexiform arrangement	Frequently encountered [24,25,26]	Dense neural interconnections within the parotid plexus
Terminal branch communication	Most commonly between buccal–marginal mandibular and buccal–zygomatic branches [24,25,26]	Functional overlap between terminal branches
Branching asymmetry	May occur between sides [22,24,25,26]	Right and left FN patterns may differ in the same individual
Landmark variability	TP demonstrates greater variability than the digastric muscle or tympanomastoid suture [22,27,28]	TP is less consistent than the digastric muscle or tympanomastoid suture
FN–adjacent nerve communication	Uncommon [29]	Connections with the auriculotemporal nerve and CN IX pathways may occur

**Table 2:** Disease-related anatomical distortion of the facial nerve.

Pathology	Primary Mechanism of Distortion	Characteristic FN Alteration	Most Vulnerable FN Region	Major Anatomical Consequence
Vestibular schwannoma	Progressive tumor expansion and capsular adhesion	FN stretching, thinning, flattening, and displacement	Cerebellopontine angle and IAM	Normal FN trajectory becomes severely distorted [30,31,32]
Hemifacial spasm	Chronic neurovascular compression	Mechanical irritation at the root exit zone	Proximal cisternal FN	Neurovascular anatomy becomes pathologically altered [33,34,35]
Temporal bone trauma	Fracture-associated compression or disruption	Edema, entrapment, or transection	Intratemporal FN	Rigid osseous anatomy limits tolerance to swelling [36]
Parotid tumors	Mass effect and branch displacement	Distortion of terminal branch orientation	Extratemporal/intraparotid FN	Complex plexiform anatomy becomes increasingly difficult to identify
Iatrogenic surgical injury	Traction, thermal injury, or branch transection	Loss of normal fascicular continuity	Intraparotid and peripheral branches	Macroscopically preserved nerve may remain functionally impaired
Aberrant postoperative regeneration	Misdirected parasympathetic reinnervation	Formation of abnormal neural connections	Auriculotemporal–FN communications	Pathological reorganization contributes to Frey syndrome [37]

**Table 3:** Anatomical determinants of facial nerve preservation across contemporary surgical procedures.

Surgical Context	Critical Anatomical Challenge	Most Important Preservation Strategy	Principal Limitation of Preservation
Vestibular schwannoma surgery	FN thinning, stretching, and capsular adhesion distort normal nerve trajectory	Atraumatic capsular dissection with continuous neurophysiological monitoring	Severe tumor adhesion may preclude safe anatomical preservation despite gross total resection goals [37,38,39,40,41,43,44]
Microvascular decompression for HS	Complex neurovascular relationships at the root exit zone	Precise identification and mobilization of the offending vessel	Persistent compression may occur despite anatomically adequate decompression [42,45,46,47,50,57]
Parotidectomy	Highly variable intraparotid branching and plexiform anatomy	Early FN trunk localization with meticulous peripheral branch dissection	Extensive branching variability limits reliability of standardized dissection planes [21,22,24,25,48,58]
FN repair and grafting	Loss of fascicular continuity and proximal-distal alignment	Early tension-free neurorrhaphy or interposition grafting	Functional recovery decreases with prolonged denervation and fibrosis [49,59,60]
Nerve transfer and facial reanimation	Absence of a functional proximal FN stump	Recruitment of alternative motor pathways through hypoglossal or masseteric transfer	Restoration of spontaneous facial expression remains limited despite reinnervation [51,52]

**Table 4:** Contemporary determinants of facial nerve preservation and postoperative functional outcomes.

Determinant	Key Influence on FN Outcome	Principal Limitation
FN displacement and adhesion	Distorted anatomy increases operative difficulty and traction-related injury risk	Severe capsular adhesion may prevent safe dissection [31,53]
Tumor consistency and stromal fibrosis	Fibrous or cystic tumors reduce dissection planes and increase manipulation	Anatomical preservation does not always predict functional preservation [53]
MRI-based preoperative mapping	Improves assessment of FN position, distortion, and tumor consistency	Accuracy decreases in severely distorted anatomy [61,62]
DTIT	Enables patient-specific estimation of FN trajectory	Reliability remains dependent on imaging quality and tumor anatomy [63]
Intraoperative neurophysiological monitoring	Provides real-time functional assessment during dissection	Monitoring supplements but does not replace anatomical expertise [65,66]
Patient-specific biological factors	Younger age and earlier intervention favor functional recovery	Recovery potential remains variable between individuals [64]
Artificial intelligence models	Integrates multidimensional surgical and imaging variables for outcome prediction	Current evidence remains limited by retrospective and non-standardized datasets [67,68]

## Data Availability

No new datasets were generated or analyzed during the current study. All data discussed in this review are derived from previously published studies.

## Abbreviations

AICA, anterior inferior cerebellar artery; AI, artificial intelligence; CN, cranial nerve; DTIT, diffusion tensor imaging tractography; EMG, electromyography; FN, facial nerve; HS, hemifacial spasm; IAM, internal acoustic meatus; LS, labyrinthine segment; LSR, lateral spread response; MRI, magnetic resonance imaging; PICA, posterior inferior cerebellar artery; TMP, tip of the mastoid process; TP, tragal pointer; VS, vestibular schwannoma.
